# The Chelsea Hospital for Women

**Published:** 1894-10-20

**Authors:** 


### The Hospital

An Institutional Journal of

The Medical Sciences and Hospital Administration, with

An Extra Nursing Supplement.

Vol. XVII.—No. 421.] [Oct. 20, 1894.

### THE CHELSEA HOSPITAL FOR WOMEN.

It is impossible to avoid an expression of extreme regret at the position taken up by the Board of Management of the Chelsea Hospital for Women. Whether or no this hospital has shown any justification for its existence, whether it is right that special hospitals should be erected for the treatment of such very wide groups of diseases as may be included under the term diseases of women, or whether it is well that money should be diverted by these special institutions from the coffers of general hospitals where exactly the same class of maladies are being efficiently treated, are questions on which we do not now propose to touch further than to draw attention to the obvious truism that unless special institutions can show better results than can be obtained at general hospitals there is no excuse for their existence, and this we fear the Chelsea Hospital for Women has failed to do.

What we do, however, feel bound to reiterate is that the management of a hospital ought to be such as to command the confidence of the public, and it is on this account that we regret the present position of the Chelsea Hospital. There can be no doubt about the facts. In consequence of reports publicly made by a legally-constituted authority as to the sanitary condition of the hospital, and as to the mortality among its patients, a Commission of Inquiry was appointed, the members of which were of such a standing as to command confidence. This Commission took evidence, and issued a report reflecting strongly on the management of the hospital and, but in somewhat different degree, upon the members of the medical staff.

Unfortunately, as one may perhaps say, no attempt was made to allocate blame to individuals, the public was left entirely in the dark as to who or how many members of the staff were considered by the Commission to have been at fault. Under such circumstances it was of the extremest importance, as a means of restoring confidence in the hospital, that the new staff, whether comprising old members or new, should be appointed by some body entirely independent of the inculpated Board of Management. The medical staff resigned, and perhaps it would have been better, certainly it would have been more seemly, had the Board done so also. Instead of which they elected a new staff, arbitrarily including some of the old members and excluding others. Is it then to be wondered at that people outside ask each other what it all means, and wonder in which group are the good men, in those admitted or in those turned out; all they know is that those admitted have been put in office by the very Board which was condemned by the Commission, which is not entirely satisfactory.

There can be but little doubt that if the Board intended ultimately to resign they should have done so at first along with the medical staff, and that they showed great want of discretion in tying the hands of their successors, first by appointing a new staff, and next by laying down a new policy, as was done in the series of proposals laid before the meeting. Out of these proposals arises another point on which a word must be said, viz., the large share taken by the medical staff in the collection of funds for the support of the institution. It is a custom not peculiar to this hospital, but it is one which in this instance has attained considerable proportions, and it is one which, if allowed to develop and extend, may ultimately become a very canker eating into the management of our charities.

Among the recommendations of the Board of Management are two which especially emphasise this point. It was proposed to confer upon the Board of Management power to suspend or dismiss any member of the medical staff, after full inquiry, if he had acted in a manner detrimental to the interests of the hospital, not merely, be it noted, for professional misdeeds; and it was also proposed that the staff should retire annually, but be eligible for re-election. This is a tremendous engine for the suppression of criticism, and we doubt whether it would be for the benefit of the hospitals of London that such a proposal should be largely copied. This appointment of the Board as absolute masters of the staff, holding over them the power of dismissal, with all the discredit which would inevitably attend it, throws up into all the stronger light the possible evils of the system of encouraging members of the staff to figure largely as collectors of funds. We believe that the medical officers of our hospitals are appointed because of their known position and recognised ability, but in the possibility of candidates being measured by Boards of Management, according to their powers of raising funds, we see a small cloud, not the size of a man's hand, but of his purse, which if not quickly and firmly dealt with may overshadow our great hospital system, greatly to the detriment of its scientific position.

In regard to other points raised at the meeting, it is probable that the position of the hospital will be definitely strengthened by the new proposals. The appointment of a Weekly Board, with a separate chairman to carry on the actual management, is a move in the right direction, but great care will be required in its selection, and it will be necessary to define its functions accurately to avoid clashing with the Board of Management, in fact with an active Weekly Board the Board of Management might well confine itself to matters of finance, letting of tenders, &c, and certainly need not meet so frequently as once a month.

There can be no doubt about the advantage of having a pathologist, not a mere nominee of the staff, but an independent officer, to be directly responsible to the Board, and, if possible, one whose aim is pathological investigation rather than work in the particular specialty to which the hospital is devoted. The appointment of the medical staff as a medical committee to advise on all professional matters is also a step in the right direction, not only because it will ensure that the Board shall not be swayed by individual opinions, and that whatever action is taken on matters touching the medical administration shall be founded on the deliberately recorded vote of the whole staff, but that the meetings of this committee will draw the staff together and enable them to deal collectively with matters which it is to be feared have hitherto been looked at from a strangely individual point of view. For we find it hard to believe that if there had been proper meetings of the whole staff it would have been possible to bring forward as an excuse for performing operations in the midst of septic conditions, the fact that the septic deaths had been distributed among different operators, and therefore did not become patent till the end of the year.

